# Characteristics of Anti-Contactin1 Antibody-Associated Autoimmune Nodopathies With Concomitant Membranous Nephropathy

**DOI:** 10.3389/fimmu.2021.759187

**Published:** 2021-10-05

**Authors:** Qianhui Xu, Shuhu Liu, Peng Zhang, Zhen Wang, Xin Chang, Yulu Liu, Jiahe Yan, Ruirong He, Xiaoguang Luo, Liang-Yu Zou, Xiaofan Chu, Yi Guo, Suli Huang, Xuejun Fu, Ying Huang

**Affiliations:** ^1^ Department of Neurology, Shenzhen People’s Hospital (The Second Clinical Medical College, Jinan University, The First Affiliated Hospital, Southern University of Science and Technology), Shenzhen, China; ^2^ Department of Research and Development, Guangzhou Weimi Bio-Tech Co., Ltd, Guangzhou, China; ^3^ Department of Nephrology, Shenzhen People’s Hospital (The Second Clinical Medical College, Jinan University, The First Affiliated Hospital, Southern University of Science and Technology), Shenzhen, China; ^4^ Department of Psychiatry, Third Affiliated Hospital of Southern University of Science and Technology, Shenzhen, China; ^5^ Department of Environment and Health, Shenzhen Center for Disease Control and Prevention, Shenzhen, China

**Keywords:** anti-Contactin 1, autoimmune nodopathies, membranous nephropathy, cidp, membranous glomerulonephritis, autoimmune neuroinflammation, demyelination

## Abstract

**Background:**

The concurrence of anti-contactin 1 (CNTN1) antibody-associated chronic inflammatory demyelinating polyneuropathy (CIDP) and membranous nephropathy (MN) has previously been reported in the literature. CIDP with autoantibodies against paranodal proteins are defined as autoimmune nodopathies (AN) in the latest research. In view of the unclear relationship between CIDP and MN, we performed a case study and literature review to investigate the clinical characteristics of anti-CNTN antibody-associated AN with MN.

**Methods:**

We detected antibodies against NF155, NF186, CNTN1, CNTN2, CASPR1 and PLA2R in blood samples of a patient with clinically manifested MN and concomitant peripheral neuropathy *via* double immunofluorescence staining and conducted a quantitative measurement of anti-PLA2R IgG antibodies *via* enzyme-linked immunosorbent assay (ELISA). Case reports of anti-CNTN1 antibody-associated AN, anti-CNTN1 antibody-associated AN with MN, and CIDP with MN were retrieved through a literature search for a comparative analysis of clinical characteristics. The cases were grouped according to the chronological order of CIDP and MN onset for the comparison of clinical characteristics.

**Results:**

A 57-year-old man with anti-PLA2R positive MN was admitted to the hospital due to limb numbness, weakness, and proprioceptive sensory disorder. He was diagnosed with anti-CNTN1 antibody-associated AN and recovered well after immunotherapy. Our literature search returned 22 cases of CIDP with MN that occurred before, after, or concurrently with CIDP. Good responses were achieved with early single-agent or combination immunotherapy, but eight out of the 22 patients with CIDP and concomitant MN ultimately developed different motor sequelae. Five patients had anti-CNTN1 antibody-associated AN with MN. Among these patients, males accounted for the majority of cases (male:female=4:1), the mean age at onset was late (60.2 ± 15.7 years, range 43–78 years), and 40% had acute to subacute onset. Clinical manifestations included sensory-motor neuropathy, sensory ataxia caused by proprioceptive impairment, and elevated cerebrospinal fluid protein levels.

**Conclusion:**

The age at onset of CIDP with MN was earlier than that of anti-CNTN1 antibody-associated AN. MN may occur before, after or concurrently with CIDP. The early detection and isotyping of anti-CNTN1 and anti-PLA2R antibodies and the monitoring of isotype switching may be essential for suspected CIDP patients.

## Introduction

Chronic inflammatory demyelinating polyneuropathy (CIDP) is an immune-mediated demyelinating neuropathy. Typical CIDP manifests as progressive, stepwise, or recurrent symmetric proximal and distal weakness and sensory disturbances, which are known to develop over a course of 2 months (1, 2). Although cell-mediated and humoral immunity likely play a role in CIDP, the etiology of the disease has not been elucidated.However, recent studies have reported that autoimmune antibodies against nodal- paranodal cell- adhesion molecules such as neurofascin-155 (NF155) (3, 4), contactin-1 (CNTN1) (5–7) and contactin-associated protein 1 (CASPR1) (8) and neurofascin isoforms (NF140/186) (9) are found among subgroups of CIDP patients. In the latest guideline (1), considering patients with these antibodies often have specific clinical characteristicand pathological manifestations, CIDP with autoantibodies against paranodal proteins are defined as Autoimmune nodopathie (AN).

The concurrence of immune diseases is commonly encountered in clinical practice (10, 11). CIDP may occur as an isolated disease or in association with other systemic diseases. The concurrent existence of CIDP and membranous nephropathy (MN), although a rare clinical phenomenon, has long been reported in case studies (12). Tests for antibodies against PLA2R and THSD7A provide a clear description of the autoimmune nature of idiopathic MN (13). In one study, an small number of CIDP patients with MN were subjected to testing for antibodies against paranodal proteins, which led to the detection of anti-CNTN1 antibody positivity (7).

CNTN1 is a cell adhesion molecule belonging to the immunoglobulin superfamily. The axonal proteins CNTN1 and CASPR1 associate with glial NF155 to form septate-like junctions to maintain ion channel clustering at the nodes of Ranvier (14). CNTN1 is regarded as fundamental for maintaining saltatory conduction; the loss of CNTN1 in genetically modified mice leads to decreased nerve conduction velocity (15). However, the role of anti-CNTN1 antibodies in concomitant MN remains unclear and there is no fixed chronology for the onset of MN and CIDP. To aid in the elucidation of the common immune mechanisms of CIDP and MN, we performed an in-depth investigation of autoantibody levels for the comparative analysis of clinical characteristics among patients suffering from CIDP with MN and anti-CNTN1 antibody-associated AN, with different chronological orders of CIDP and MN onset.

## Materials and Methods

### Case Presentation

**Figure 1:**
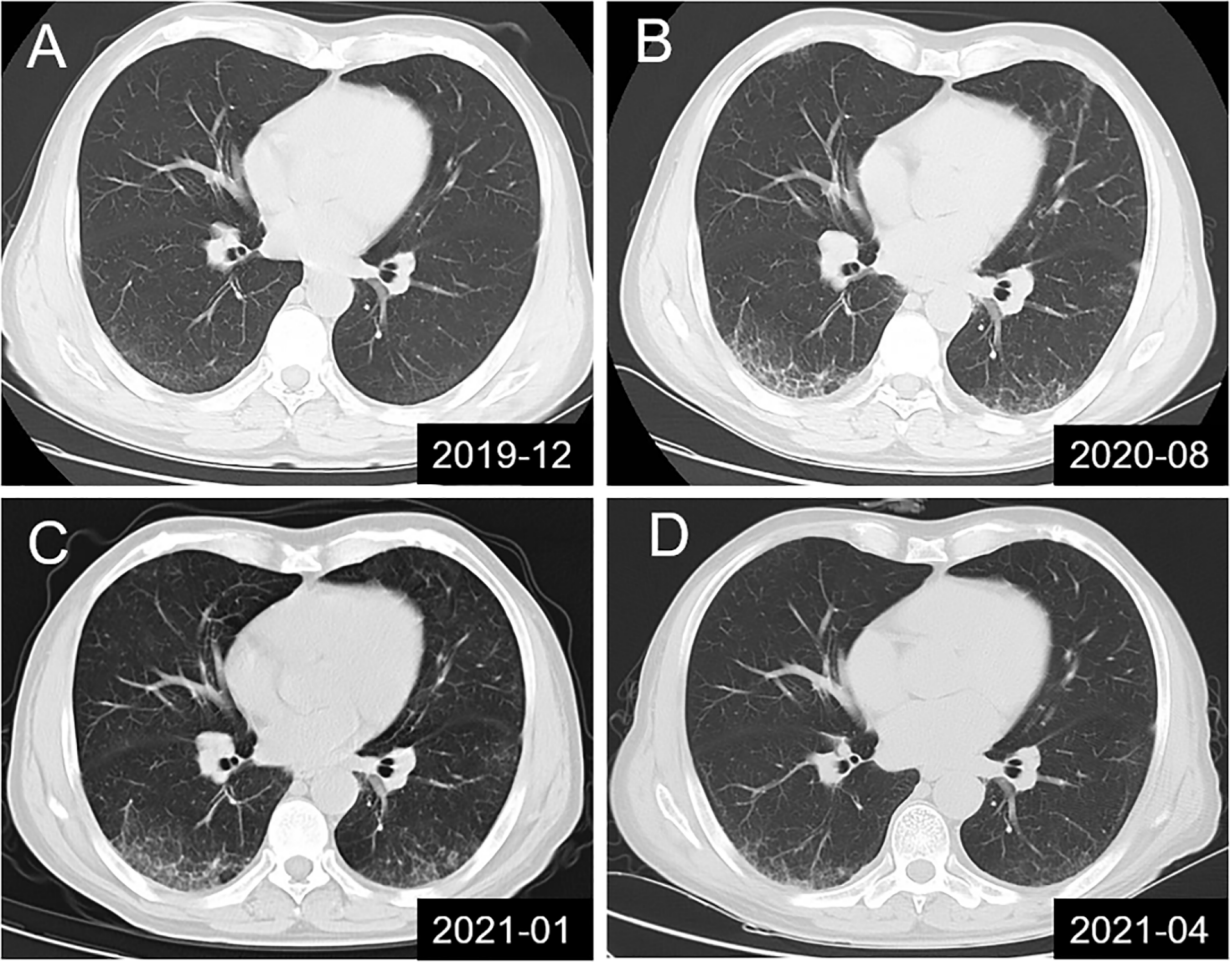
Lung CT scans showing dynamic changes in interstitial pneumonia in the studied patient. **(A)** Mild interstitial pneumonia found during physical examination; **(B, C)** Dynamic monitoring before treatment and in the absence of clinical symptoms; **(D)** Significant improvement in interstitial pneumonia was observed in the results of lung CT re-examination at 10 days after combination therapy with intravenous immunoglobulin, methylprednisolone and rituximab.

A 57-year-old man sought medical attention at our hospital after experiencing progressive limb weakness and sensory abnormalities for six weeks. The patient had developed nephrotic syndrome three years earlier. The results of his renal biopsy suggested the presence of PLA2R-associated MN and fulfilled the criteria for stage 3 MN, but immunotherapy had not yet been administered. Results of the initial physical examination at admission indicated symmetric limb weakness with Grade V^-^ muscle strength in all four limbs; strength was impaired in both proximal and distal muscles in the upper and lower limbs, poor stability and accuracy in the finger-to-nose and heel-to-knee-to-shin test, loss of tendon reflexes, decreased proprioception, and decreased distal superficial sensation in a glove and stocking pattern. The patient required mobility aids for walking. Neuroelectrophysiological results were as follows: motor distal latency prolongation ≥50% above the upper limit of normal values (ULN) in the bilateral peroneus and right medianus; reduction of motor conduction velocity ≥30% below the lower limit of normal values (LLN) in the bilateral medianus, right musculocutaneous nerve, femoral nerve, and left peroneus; motor conduction block, ≥30% reduction of the proximal relative to distal negative peak compound muscle action potential (CMAP) amplitude in the bilateral medianus, right ulnaris, and left peroneus; sensory conduction abnormalities (prolonged distal latency or reduced sensory nerve action potential [SNAP] amplitude or slowed conduction velocity outside of normal limits) in the medianus, ulnaris, radial nerve, peroneus supers, and suralis. This was mainly manifested in the distal lower limbs (**Table 1**), which fulfilled the electrophysiological diagnostic criteria for CIDP developed by the Federation of European Neuroscience Societies/Peripheral Nerve Society (1). Results of the routine cerebrospinal fluid (CSF) biochemical analysis were as follows: chloride, 132.2 mmol/L; glucose, 3.00 mmol/L; protein, 1.65 g/L; and total cell count, 7/uL (nucleated cell count: 7/uL). Other biochemical test results were as follows: serum albumin, 29.8 g/L; triglycerides, 1.09 mmol/L; total cholesterol, 4.75 mmol/L; high-density lipoprotein, 1.03 mmol/L; and low-density lipoprotein, 2.85 mmol/L. The serum PLA2R antibody titer measured by ELISA was 23.75 RU/mL (negative: <14.00 RU/mL). Abnormalities were not found in the anti-NMDAR, anti-AMPAR1, anti-AMPAR2, anti-LGI1, anti-CASPR-2, and anti-GABABR antibodies in CSF. Tests for paraneoplastic antibodies (Hu, Yo, Ri, CV2/CRMP5, Ma1, Ma2, SOX1, Tr, Zic4, GAD65, PKCγ) and anti-ganglioside antibodies (GM1, GM2, GM3, GM4, GD1a, GD1b, GD2, GD3, GT1a, GT1b, GQ1b) in the serum were negative. Abnormalities were not found in complement and immunoglobulin levels and monoclonal immunoglobulins were not detected on immunofixation electrophoresis. Lung CT revealed the presence of interstitial pneumonia (**Figures 1A–C** show pre-treatment CT scans, **Figure 1D** shows the post-treatment scan). No obvious abnormalities were observed in the cervical and lumbar spine MRI results. 

**Table 1:** Dynamic changes in electromyography (EMG) before and after treatment.

	Normal	Case presentation	Case presentation after treatment
Time from onset		6 weeks	4 months
Side		L/R	L/R
**Median nerve**			
Distal latency (ms)	<4.2	6.46/8.17	3.92/3.65
MCV (m/s)	>48	33.4/36.2	47.4/40.9
CMAP amplitude (mV)	>5.0	5.5/1.18	10.2/7.7
F wave latency (ms)	<31	33.3/NR	41.3/41.7
F wave response frequency		10%/NR	55%/45%
SCV (m/s)	>44	NR/17.5	34.4/31.4
SNAP amplitude (µV)		NR/3.8	5.2/4.7
**Ulnar nerve**			
Distal latency (ms)	<3.4	6.61/5.25	3.81/3.23
MCV (m/s)	>49	39.5/41.6	48.1/46.1
CMAP amplitude (mV)	>5.0	5.4/9.5	6.7/11
F wave latency (ms)	<32	31.1/32.6	39.8/38.0
F wave response frequency		30%/45%	100%/90%
SCV (m/s)	>44	NR/NR	40.1/40.2
SNAP amplitude (µV)		NR/NR	3.9/4.4
**Tibial nerve**			
Distal latency (ms)	<6.0	5.3/6.39	4.75/4.69
MCV (m/s)	>41	30.4/28.8	39.4/36.2
CMAP amplitude (mV)	>4.8	3.5/4.4	7.6/6.1
F wave latency (ms)	<58	NR/NR	70.0/70.5
F wave response frequency		NR/NR	63.2/75
H reflex		NR/NR	NR/NR
**Sural nerve**			
SCV (m/s)	>45	43.3/49	44.2/51.1
SNAP amplitude (µV)		10.6/7.6	10.6/5.2

### Cell-Based Assays for PLA2R, NF155, NF186, CNTN1, CNTN2, and CASPR1 antibodies

In order to identify PLA2R, NF155, NF186, CNTN1, CNTN2, and CASPR1 antibodies, we subcloned each human cDNA (accession number: NM_007366, NM_001160331, NM_001005388, NM_001843, NM_005076, NM_003632) into pcDNA3.1-mCherry and transfected with HEK293 cells respectively. Twenty-four hours after transfection, live HEK293 cells were fixed with 4% paraformaldehyde for 15 minutes and permeabilized with 0.3% Triton X-100 for 5 minutes. Cells were then incubated for 1 hour at 37°C with patients’ serum (1:10), followed by the corresponding fluorescent secondary antibodies (Alexa Fluor 488 goat anti-human IgG, Invitrogen A11013; Dilution 1:500). Reactivity was determined using a fluorescence microscope by two investigators independently.

### Determination of Patients’ Antibody Titers and IgG Subclasses

Antibody titers were determined with CBA using serial dilutions of patients’ serum (dilution range 1:10 to 1:1000): the titer was defined as the highest dilution for which the reactivity with HEK293 cells was still visible. IgG subclass of patients’ antibodies was determined with PLA2R or CNTN1 CBA using fluorescent secondary antibodies specific for IgG1, IgG2, IgG3 or IgG4 (mouse anti-human,Sigma F0767,F4516,F4641 or F9890).

### Literature Review

We searched PubMed for reports of CIDP with MN using the keywords “CIDP,” “Autoimmune nodopathies,” “membranous nephropathy,” and “membranous glomerulonephritis”. Our literature search identified and included 19 articles (7, 12, 16–32) describing 22 cases of CIDP with MN, of which five cases were of anti-CNTN1 antibody-associated AN with MN (including the present case). A study recorded one patient with anti-CNTN1 IgG3/IgG4 antibodies who had a concurrent onset of CIDP and MN. This study was not included in the analysis due to lack of clinical data (33).

## Results

### Results of Renal Biopsy and Immunohistochemical Testing

**Figure 2:**
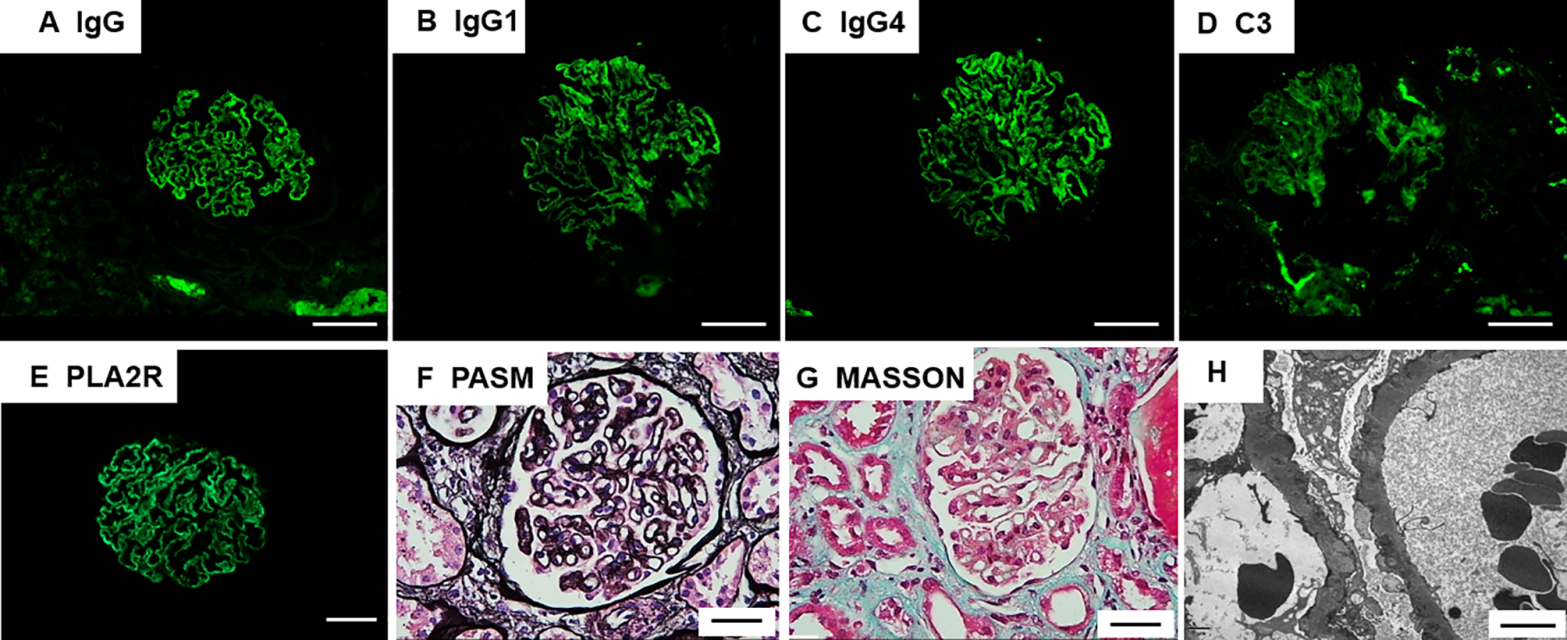
Renal pathology findings. **(A)** Immunofluorescence microscopy demonstrates granular deposits of IgG along the glomerular basement membrane; Scale bar = 20µm **(B, C)** IgG1 and IgG4 deposits detected by IgG subclass staining; Scale bar = 20µm **(D)** C3 deposits shown by immunohistochemical staining; Scale bar = 20µm **(E)** Immunostaining for phospholipase A2 receptor (PLA2R) is positive; Scale bar = 20µm **(F)** Periodic acid-methenamine silver (PAM) staining of the patient’s glomeruli reveals a spiked appearance of the glomerular basement membrane; Scale bar = 20µm **(G)** Masson’s trichrome staining revealed fuchsinophilic protein deposits beneath the visceral layer; Scale bar = 20µm **(H)** Non-uniform diffuse thickening of the glomerular basement membrane, diffuse foot process effacement, and abundant electron-dense deposits beneath the epithelium and within the basement membrane, with a portion of the electron-dense matter embedded and absorbed in the basement membrane. Scale bar = 4µm.

The results of immunofluorescence staining were as follows: IgG (+++), IgA (-), IgM (+), C3 (+), C1q(-), Fib(-), ALB (reabsorption droplets were observed in the renal tubular epithelial cells), kappa(+), lambda(+), IgG1(++), IgG2(+), IgG3(-), IgG4(++), PLA2R(+++), and THSD7A(-). Immunofluorescence microscopy revealed that deposits containing granular IgG and PLA2R (+++) were along the capillary walls. Ultrastructural examination revealed non-uniform diffuse thickening of the glomerular basement membrane, diffuse foot process effacement, and abundant electron-dense deposits beneath the epithelium and within the basement membrane, with a portion of the electron-dense matter embedded and absorbed in the basement membrane (**Figure 2**).

### Detection of Antibodies and Antibody Subclasses

**Figure 3:**
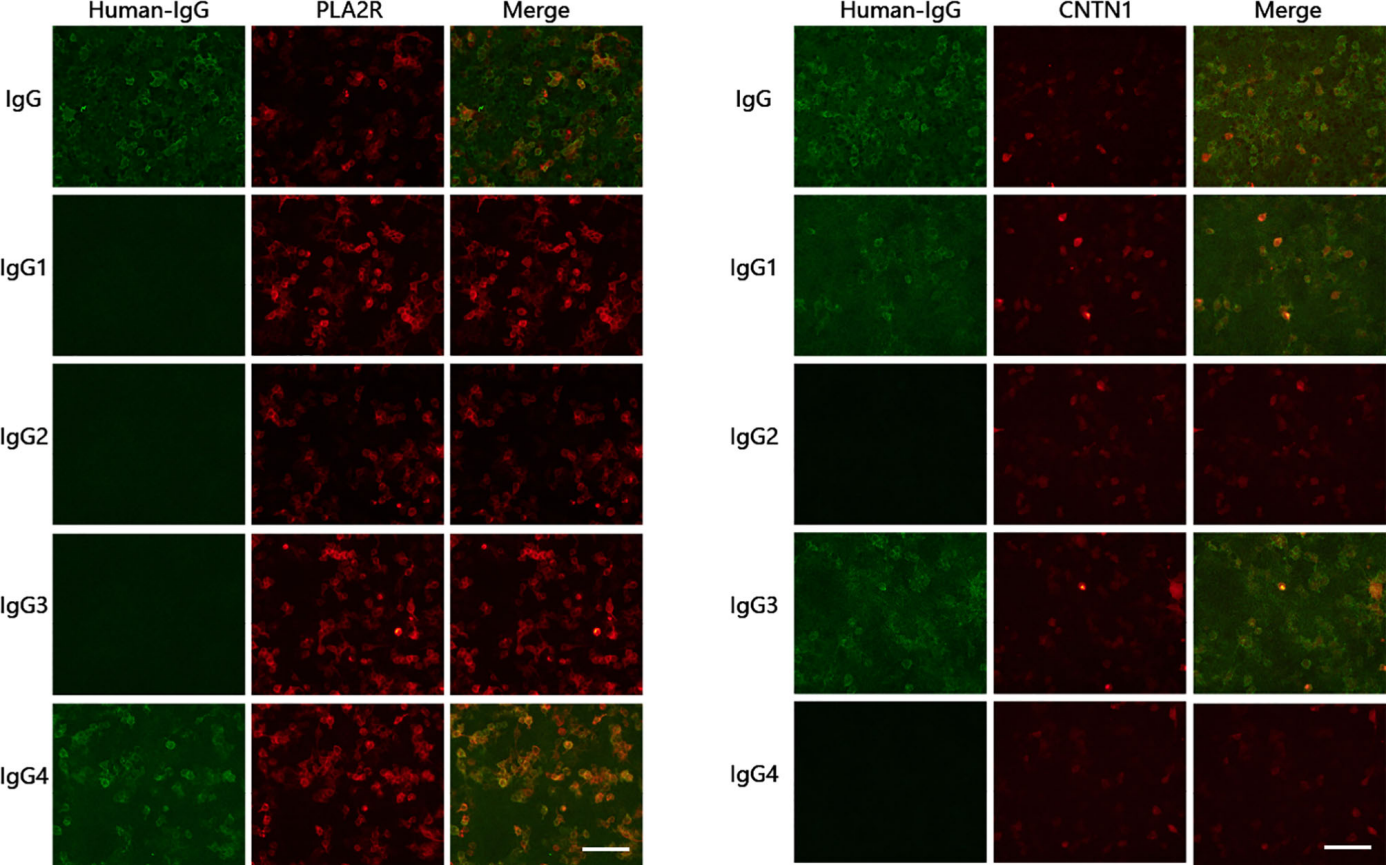
Detection of anti-CNTN1 and anti-PLA2R antibodies and subclasses. Anti-CNTN1 antibody subclasses: IgG3(+), IgG1(+); serum anti-PLA2R antibody subclasses: IgG4(+). Scale bar = 100 µm.

Antibodies associated with the nodes of Ranvier in serum samples included a positive finding for the anti-CNTN1 antibody (1:300) and negative findings for all other antibodies (anti-NF155, anti-NF186, anti-CNTN2 and anti-CASPR1 antibodies). Subclasses for anti-CNTN1 antibody included IgG3(+) and IgG1(+); subclasses for the serum anti-PLA2R antibody were comprised solely of IgG4(+) (**Figure 3**).

### Diagnosis and Treatment Outcomes

Through clinical manifestations, EMG results and antibody testing, the patient was diagnosed as anti-CNTN1 antibody-associated AN. Based on previous treatment experiences, the patient was administered with pulse therapy of immunoglobulin (IVIG, 0.4 g/kg) and methylprednisolone (1 g/day) for 5 days, after which the patient showed improvement in limb weakness. In view of the presence of concomitant MN, a single dose of rituximab (650 mg) was administered. The patient was able to walk without the use of mobility aids after approximately 10 days and could walk normally at the one-month follow-up. Substantial improvements were observed in interstitial pneumonia, as shown by the results of lung CT re-examination at 10 days after treatment (**Figure 1D**) and the results of EMG re-examination at the four-month follow-up (**Table 1**).

### Clinical Characteristics of Anti-CNTN1 Antibody-Associated AN With MN

Five cases of anti-CNTN1 antibody-associated AN with MN (including the present case) have been reported in the literature. Our patient was the first to develop MN prior to exhibiting anti-CNTN1 antibody positivity. The predominant IgG subclass of the majority of cases was IgG4; the predominant subclass in our case was IgG3 (**Table 2**). The mean age at onset was 60.2 ± 15.7 years (range 43–78 years, 40% of patients >60 years) and the male/female ratio was 4:1. Disease onset was chronic in three cases and acute in 1 case and subacute onset in 1 case. Proprioceptive impairment or sensory ataxia was manifested in three patients (60%). The mean CSF protein level was 196 ± 125 mg/dL (range61–400 mg/dL), with levels >100 mg/dL occurring in four patients (80%). Most patients initially responded well to immunotherapies such as corticosteroids (CS), plasma exchange (PE), and intravenous immunoglobulin (IVIg) (**Table 3**).

**Table 2:** Clinical Characteristics of anti-CNTN1 antibody-associated AN with MN.

Course/time	Country/time	Author	Age/sex	Motor functions at peak of illness	Period from onset to peak of CIDP	Sensory ataxia or disturbed deep sensation	24-hour urine protein	CSF protein levels (mg/dL)	Anti-CNTN1 antibodies	Serum antibodies	Renal biopsy	Treatment and response of CIDP	Treatment and response of MN
CNTN1 -MN/concurrent	Germany/2015	Doppler et al. (7)	48/M	Tetraparesis	Acute-like GBS	ND (severe sensory disturbance)		204mg/dL	IgG4>IgG3>IgG2>IgG1	ND	ND	IVIg, initial improvement; CS, transiently improved; PE, improved	Complete recovery (treatment efficacy was not well documented)
CNTN1 - MN/concurrent	Japan/2018	Hashimoto Y et al. (25)	78/F	Unable to walk without help, Weakness that was more marked in the legs than in the arms	>2 months	Sensory ataxia	4.1g/24 h	61 mg/dL	IgG4 and IgG1 were dominant	Anti-SSA/Ro (1:16), Anti-PLA2R(-), anti-THSD7A (-)	Stage 2 IgG1(-), IgG2(-), IgG3(-), IgG4(+), PLA2R (weakly positive)	CS, ineffective; IVIg, improved, able to walk at 2 months after treatment	CS, improved
CNTN1 - MN/5M	France/2019	Guillaume Taieb et al. (26)	75/M	Wheelchair, Strength was 2/5 proximally and distally in lower limbs and 3/5 distally in upper limbs.	5M	Romberg sign(+),Vibratory loss,	5.4 g/24 h	150 mg/dL	IgG4(+)	Anti-PLA2R(-), NF-155 and NF-186(-)	IgG4(+), C3(+), C1q(-), PLA2R(-).	Prednisone + PE + rituximab, worsened, died from aspiration pneumonia	Prednisone, improved
CNTN1 - MN/12M	Canada/2020	Nazarali, S et al. (32)	43/M	wheelchair dependency	4M	ND	22 g/h	400 mg/dL	IgG4(+)	Anti-PLA2R(-)	Stage 2 MN, IgG(+), CNTN1(+)	IVIG + prednisone, good clinical and electro-physiologic response	Cyclosporin + prednisone, good
MN - CNTN1/3Y	China/2021	Our case	57/M	wheelchair dependency	6 weeks	Sensory ataxia, Decreased proprioception	7.8g/24h	160 mg/dL	IgG1(+), IgG3(+), IgG2(-), IgG4(-)	Anti-PLA2R(+), NF-155 and NF-186(-)	Stage 3 MN IgG3(-), IgG4(+); PLA2R(+); THSD7A(-)	Rituximab + hormones + γ-globulin, good	Rituximab + hormones + γ-globulin, good

**Table 3:** Comparison of the clinical characteristics of chronic inflammatory demyelinating polyneuropathy (CIDP) with membranous nephropathy (MN), anti-contactin 1 (CNTN1) antibody-positive AN with MN, and anti-CNTN1 antibody-associated AN.

Characteristic	CIDP with MN (n=22)	anti-CNTN1 antibody-associated AN with MN (n=5)	anti-CNTN1 antibody-associated AN (n=20)
Male/female ratio	17:5 (3.4:1)	4:1 (4:1)	14:6 (2.3:1)
Age at onset of CIDP (mean ± SD, years)	49.3 ± 19.3 (range 9–81)	60.2 ± 15.7 (range 43–78)	63 ± 13.5 (range 33–81)
Onset age of CIDP >60 years	6 (27.3%)	2 (40%)	16 (80%)
**Mode of onset^a^**			
Acute onset	4 (18.2%)	1 (20%)	7 (35%)
Subacute onset	2 (9.1%)	1 (20%)	3 (15%)
Chronic onset	16 (72.7%)	3 (60%)	10 (50%)
Sensorimotor neuropathy	19 (86.4%)	4 (80%)	19 (95%)
Distal dominant muscle weakness	13 (59.1%)	0	14 (70%)
Proprioceptive impairment or sensory ataxia	16 (72.7%)	3(60%)	15 (75%)
CSF protein level (mean ± SD, mg/dl)	278 ± 280 (range 61–1320)	196 ± 125 (range 61–400)	253 ± 1432 (range 79–693)
CSF protein>100 mg/dl^b^	18 (81.8%)	4 (80%)	19 (95%)
**Efficacy of immunotherapies for CIDP**			
CS	13/20 (65%)	3/5 (60%)	5/17 (29%)*
PE	3/8 (37.5%)	1/2 (50%)	5/7 (71%)
IVIg	6/11 (54.5%)	4/4 (100%)	4/7 (57%)**
Combination^c^	6/9 (66.7%)	2/3 (66.7%)	ND

^a^Defined as acute for less than 1 month, subacute for 1-2 months, and chronic for more than 2 months.

^b^Two values were missing for CSF protein level.c：Combination immunotherapies included PE+methotrexate, PE+CS+azathioprine, PE+CS+IVIg, PE+CS+IVIg+cyclosporine, and hormones+cyclophosphamide.

*Partial or transient response is regarded as ineffective.

**Initial improvement in acute onset cases is counted as effective.

### Comparisons of Characteristics of CIDP With MN and Anti-CNTN1 Antibody-Associated AN

We identified reports of 22 cases of CIDP with MN (**Table 3**). The mean age at onset was 49.3 ± 19.3 years (range 9–81 years, 27.3% of patients were >60 years) and the male/female ratio was 17:5 (3.4:1). Disease onset was chronic in 16 cases, acute (≤1 month) in 4 cases, and subacute (1-2 month) in 2 cases. All patients had typical CIDP. Distal dominant symmetric limb weakness was manifested in 13 cases (59.1%), and proprioceptive impairment or sensory ataxia was manifested in 16 cases (72.7%). The mean CSF protein level was 278 ± 280 mg/dL (range61–1320 mg/dL), with levels >100 mg/dL occurring in 18 patients (81.8%). Efficacies of the immunotherapies were as follows: CS, 13/20 (65%); PE, 3/8 (37.5%); and IVIg, 6/11 (54.5%). These results indicate that the majority of patients (18/22, 81.8%) initially responded well to CS, PE, and IVIg. However, combination therapy consisting of three or more types of immunotherapy was ineffective in four cases (18.2%).

When the literature was re-surveyed, we found that 20 cases of anti-CNTN1 antibody-associated AN were reported in three case series studies (5–7) (**Table 3**). The mean age at onset was 63.0 ± 13.5 years (range 33–81 years, 80% were >60 years) and the male/female ratio was 14:6 (2.3:1). Disease onset was chronic in 10 cases, acute in seven cases, and subacute in three cases. Common clinical manifestations included symmetric presentations and distal dominant involvement (90%), especially sensation disorders, dominant sensory ataxia, progressive disease course, and poor response to CS and IVIg. The mean CSF protein level was 253 ± 143 mg/dL (range79–693 mg/dL, >100 mg/dL in 95% of patients).

Based on the results described above, the common characteristics of CIDP with MN and anti-CNTN1 antibody-associated AN were as follows (**Table 3**). Patients were predominantly male, acute onset occurred in a certain proportion of patients (18.2% and 35%), age at onset was relatively late, the majority of patients experienced distal sensorimotor nerve involvement and sensory ataxia caused by proprioceptive impairment, and CSF protein levels were high. Some patients with CIDP and MN were young individuals <30 years, and patients with CIDP and MN generally responded better to immunotherapy than patients with anti-CNTN1 antibody-associated AN.

### Comparison of the Characteristics of CIDP With MN With Different Chronological Orders of Onset

CIDP onset occurred first in 12 patients, both CIDP and MN onset occurred concurrently in seven patients, and MN onset occurred first in three patients (**Table 4**). Patients whose onset of MN occurred earlier than CIDP were younger (<60 years) and were more susceptible to acute onset, sensorimotor neuropathy, and proprioceptive involvement. A smaller proportion of patients with concurrently occurring CIDP and MN had sequelae. Patients with an earlier onset of MN or concurrent onset of CIDP and MN showed greater improvement in proteinuria.

**Table 4:** Comparison of the clinical characteristics of chronic inflammatory demyelinating polyneuropathy (CIDP) with membranous nephropathy (MN) with different chronological orders of onset.

Characteristic	CIDP→MN (12 cases)	MN→CIDP (three cases)	CIDP and MN occurred concurrently (seven cases)
Male/female ratio	10:2 (5:1)	3:0	4:3 (1.3:1)
Age at onset of CIDP (mean ± SD, years)	50.6 ± 20.0 (range 18–81)	33.0 ± 33.9 (range 9–57)	51.9 ± 18.2 (range 27–78)
Onset age of CIDP >60 years	3 (25.0%)	0	3 (42.9%)
Mode of onset			
Acute onset	2 (16.7%)	1 (33.3%)	1 (14.3%)
Subacute onset	0	1 (33.3%)	1 (14.3%)
Chronic onset	10 (83.3%)	1 (33.3%)	5 (71.4%)
Sensorimotor neuropathy	10 (83.3%)	3 (100%)	6 (85.7%)
Distal dominant muscle weakness	8 (66.7%)	1 (33.3%)	4 (57.1%)
Proprioceptive impairment or sensory ataxia	7 (58.3%)	3 (100%)	6 (85.7%)
CSF protein level (mean ± SD, mg/dL)	363 ± 354 (range 64–1320)	188 ± 33 (range 165–212)	170 ± 93 (range 61–350)
CSF protein>100 mg/dL	10 (90.9%)	2 (100%)	6 (85.7%)
**Efficacy of immunotherapies for CIDP**			
CS	6/11	2/3	5/6
PE	1/6	0	2/2
IVIg	2/5	2/3	2/3
Combination	3/6	2/2	1/1
Improved CIDP outcome	8/12	3/3	7/7
Motor sequelae	6/12	2/3	0/7
**Improvement of MN**			
Reduction of proteinuria	5/12	3/3	6/7

## Discussion

Most often, the course of CIDP is steadily progressive or relapsing–remitting for more than 8 weeks. Some patients (up to 13% of patients) who are eventually diagnosed with CIDP may present acute symptoms (acute-onset CIDP [A- CIDP]). Generally, patients with A-CIDP are still able to walk independently, are less likely to have facial weakness, respiratory or autonomic nervous system involvement, and are more likely to experience sensory impairment. Although these features may be beneficial for the diagnosis of A-CIDP, there are no specific clinical features or laboratory tests that can distinguish Guillain–Barre syndrome (GBS) from A-CIDP in the acute phase of the disease. However, considering its unique clinical and pathological characteristics, the latest guidelines consider AN to be an independent disease, different from CIDP (28). In our case, the patient was admitted to the hospital in the sixth week of the disease course, and the clinical manifestations continued to progress without remission. The first electromyogram result was in line with the diagnostic criteria of CIDP. In addition, the patient had a subacute onset, combined with tremor, ataxia disproportionate to the sensory involvement, associated nephrotic syndrome, and very high CSF protein levels, which were in line with the guidelines recommended for antibody testing (28). The final diagnosis of this patient was anti-CNTN1 antibody-associated AN. However, to be consistent with the analysis of the previous article, we categorized the diagnosis as CIDP.

The association of inflammatory neuropathies and nephrotic syndrome was reported for the first time in 1973 as GBS with acute glomerulonephritis (11), and the relationship between CIDP and MN was first reported by Witte and Burke in 1987 (12). However, later case reports describing the concurrence of the two diseases have been extremely rare. The case presented herein is the fifth case of anti-CNTN1 antibody-associated AN with MN in the literature and the first in which MN onset occurred prior to the exhibition of anti-CNTN1 IGg3 antibody positivity. The reported frequencies of occurrence of anti-PLA2R and anti-THSD7A antibodies in MN are 50–80% and 5–10% (13), respectively; the target antigen of MN is uncertain in 10–20% of cases. The presence of one type of antibody does not exclude the presence of other antibody types, as shown by a reported case of anti-PLA2R- and anti-THSD7A-positive MN (34). It has been suggested that MGN is secondary to an increase in serum IgG4 concentration, which may be due to the deposition of the circulating immune complexes or other unknown mechanisms. Another possibility is that CNTN1 IgG4 antibody specifically affects podocyte structure and PLA2R immunostaining (26). Despite anti-PLA2R antibody positivity, the participation of other antibodies cannot be ruled out as renal biopsies performed in previous studies have shown extensive CNTN1 staining within the glomerulus (32) and weak mRNA expression in the kidneys (35). It may be worthwhile to search for other target renal antigens of CIDP with MN, such as CNTN1.

Interestingly, our patient had concomitant interstitial pneumonia in addition to CIDP with MN, which improved after the administration of immunotherapy. Studies have shown that anti-PLA2R antibodies are not merely expressed on podocytes in normal glomeruli (36) but are also present in the lungs (37) and on leukocytes (38). Further research will be required to determine if the onset of interstitial pneumonia is associated with anti-PLA2R antibodies.

In the majority of patients with CIDP and concomitant MN, the onset of the two diseases occurred in succession within a span of two months. This may be suggestive of a common antigenic target (25). M-type PLA2R may be a possible candidate, but it is currently unclear whether PLA2R is expressed in peripheral nerves. Studies have shown that serum anti-PLA2R antibody positivity does not always occur in CIDP with MN. Other possible target antigens are NF-186 and contactin-1, with antibodies present in the nodes and paranodes of myelinated axons and expressed in the podocytes (35, 39). Researchers have deduced that the anti-contactin-1 and anti-NF-186 autoantibodies can promote changes in the glomerulus and in paranodes (26). Biopsies have shown extensive CNTN1 staining within the glomerulus (32), and low-level CNTN1 mRNA expression has been detected in healthy renal cells (35). In the present case study, the time interval between the onset of MN and CIDP was three years, which is significantly different from that of previously described cases. This suggests that our case may not be supportive of the results of the molecular simulation.

The patient in our case study only exhibited anti-CNTN1 antibody-associated AN three years after the confirmed diagnosis of MN. Sustained anti-PLA2R antibody positivity may have triggered epitope spreading, leading to the aggravation of MN or even inducing intermolecular spreading of new epitopes, resulting in CNTN1 exposure. Epitope spreading is a phenomenon in which epitopes within the same molecule or a different molecule are recognized by T or B cells from an original noncross-reactive antigenic site (40). As described in autoimmune diseases such as human anti-glomerular basement membrane disease (41), pemphigus vulgaris (42), rheumatoid arthritis (43), multiple sclerosis (44) and type I diabetes (45), intramolecular epitope spreading is associated with disease worsening. Epitopes have been identified in three domains of PLA2R1, namely CysR, CTLD1 and CTLD7. Several common mutations associated with idiopathic MN have been localized to these domains. CysR is the major dominant epitope, with evidence showing the presence of epitope spreading to CTLD1 and CTLD7. Anti-PLA2R1 reactivity against CysR at serum sampling has been associated with favorable outcomes, while reactivity against CTLD1 and CTLD7 is associated with active disease and poor renal prognosis (40).

Antibody class switching is a common phenomenon during the disease course of immune-related diseases. For instance, a switch in predominant antibody subclass from IgG1 to IgG4 occurred in multiple sclerosis patients treated with glatiramer acetate (46). IgG subclass switching has been described in membranous glomerulonephritis, with IgG1 and IgG4 respectively predominant during acute and chronic phases (47). In the present study, the results of renal biopsy performed during early disease stages indicated IgG1(++) and IgG4(++), while the serum anti-PLA2R antibody re-test during hospitalization revealed IgG4 dominance (which is characteristic of the chronic phase). Patients with anti-CNTN1 antibody-associated AN exhibited IgG1 and IgG3 subclass dominance during the acute phase and IgG4 dominance during the chronic phase (6, 7). IgG3 and IgG1 were present in our patient during the acute phase, but antibody subclasses were not measured during the chronic phase. Patients with acute-onset contactin-1-associated neuropathy may experience a switch in predominant antibody subclass from IgG3 to IgG4, but further longitudinal studies are required for confirmation. The dominance of IgG3 during the initial stage of disease may explain the initial patient response to IVIg. A switch to IgG4 may explain the poor response to immunotherapy during the later stages. This is in line with the viewpoint that patients with IgG4 autoantibodies respond poorly to IVIg treatment, as IgG4 does not induce complement activation (3).

When comparing clinical characteristics of patients with CIDP and MN, both anti-CNTN1 antibody-associated AN and MN were more common in men. A higher proportion of patients with anti-CNTN1 antibody-associated AN and concomitant MN were male (4:1). Patients whose onset of MN occurred before CIDP were younger (<60 years) and more susceptible to acute onset, sensorimotor neuropathy, and proprioceptive involvement. Ataxia is common in CIDP. Although contactin-1 can bind directly to the cerebellar structure (8), the result of anti-CNTN1 antibody testing in CSF was negative, supporting the view that ataxia is caused by proprioceptive impairment rather than cerebellar lesions. A smaller proportion of patients with concurrently occurring CIDP and MN had sequelae. Patients with an earlier onset of MN or concurrent onset of CIDP and MN showed greater improvement in proteinuria. This may be related to the adoption of more aggressive treatment approaches for CIDP with MN. Results of our literature survey indicate that acute onset and Subacute onset was common in patients with anti-CNTN1 antibody-associated AN and concomitant MN (40%), which may serve as a valuable indication for clinical diagnosis.

Myelin sheath damage was the major EMG finding of most anti-CNTN1 antibody-associated AN patients. Labasqueet et al. (48) showed that the anti-CNTN-1 antibodies of patients prevented adhesive interaction between CNTN-1/CASPR and NF-155 and induced alteration of paranodal junctions in myelinated neuronal culture. In another study, severe destruction of paranodal and nodal architecture was detected in the peripheral nerves of patients with CNTN-1-associated neuropathy, with a loss of CASPR or the nerve excitation immune response and an increase in internode length occurring at certain nodes. As the space between myelin terminal loops and axolemma widens, it may induce nerve conduction abnormalities that fulfil the electrodiagnostic criteria for CIDP. The demyelination may be, or at least partly, a secondary change related to the disturbances in axon–Schwann cell interactions resulting from axoglial detachment. Paranodal dissection and typical macrophage-mediated demyelination are characteristic features of these patients. Impairment of saltatory conduction and axonal damage may be caused by axon glial cell detachment (2). These results suggest that pathogenesis is associated with contactin-1, thereby resulting in nerve conduction disorder (8). Our findings indicate the presence of conduction disorder in the majority of patients. In certain patients, clinical manifestations improved after treatment, but this was accompanied by the aggravation of EMG manifestations. However, our patient achieved improvement in both clinical and EMG manifestations.

The majority of patients with CIDP and concomitant MN (18/22, 81.8%) initially responded well to immunotherapies such as CS, PE and IVIg, but combination therapy consisting of three or more types of immunotherapies was ineffective in four cases (18.2%). Despite these observations, issues exist in the sole use of case report reviews for the evaluation of treatment effectiveness. Although both CIDP and MN respond to immunotherapy, the supporting evidence for immunotherapy selection is inconsistent. IVIg is equally effective to high-dose prednisolone in the treatment of CIDP but is not the first-line drug for the treatment of nephrotic syndrome. In a previously reported case of anti-contactin-1-associated membranous glomerulonephritis, nephrotic syndrome was alleviated after hormone therapy, whereas neuropathy rapidly worsened (7, 26), resulting in axonal involvement. Other researchers have shown that the sole use of IVIg for the treatment of nephrotic syndrome is inadequate (32). As CIDP with concomitant MN may be heterogenous, the determination of anti-CNTN1 and anti-PLA2R IgG subclasses is necessary. In the present study, the predominant subclasses for the anti-CNTN1 and anti-PLA2R antibodies were IgG3 and IgG4, respectively. Considering that IgG4 may respond poorly to immunoglobulin treatment, we adopted combination immunotherapy (CS+IVIg+rituximab) for the simultaneous treatment of peripheral neuropathy and nephrotic syndrome. This strategy led to rapid clinical effects and the achievement of consistent improvement in both clinical and EMG manifestations. Given that a substantial proportion of patients with anti-CNTN1 antibody-associated AN and concomitant MN exhibited neurological sequelae (8/22, 36.3%) and that IgG subclasses differ across antibodies, earlier and more aggressive comprehensive treatment may be explored.

In conclusion, the age at onset of patients with CIDP and concomitant MN was earlier than that of patients with anti-CNTN1 antibody-associated AN. MN onset may occur before, after or concurrently with CIDP onset. For patients with suspected CIDP, especially young individuals with sensorimotor neuropathy and proprioceptive impairment, testing and subclass determination of anti-CNTN1 and anti-PLA2R antibodies and the monitoring of antibody class switching may be necessary even when satisfactory short-term treatment effects have been obtained with immunotherapy.

## Data Availability Statement

The raw data supporting the conclusions of this article will be made available by the authors, without undue reservation.

## Ethics Statement

The studies involving human participants were reviewed and approved by Medical Ethics Committee of Shenzhen People’s Hospital (ID: LL-KY-2021647). The patients/participants provided their written informed consent to participate in this study. Written informed consent was obtained from the individual(s) for the publication of any potentially identifiable images or data included in this article.

## Author Contributions

QX wrote the manuscript, literature retrieval and utilization and contributed to the figures and table and helped in the diagnostic process. SL detected the related antibodies. PZ, ZW, XC, YL, JY, RH were involved in care of the patients. XL, L-YZ, XFC, YG, SH, and XF revised the manuscript for intellectual content. YH helped in the diagnostic process, supported the interpretation as well as critically revising the manuscript. All authors contributed to the article and approved the submitted version.

## Conflict of Interest

Author SL was employed by company Guangzhou Weimi Bio-Tech Co., Ltd.

The remaining authors declare that the research was conducted in the absence of any commercial or financial relationships that could be construed as a potential conflict of interest.

## Publisher’s Note

All claims expressed in this article are solely those of the authors and do not necessarily represent those of their affiliated organizations, or those of the publisher, the editors and the reviewers. Any product that may be evaluated in this article, or claim that may be made by its manufacturer, is not guaranteed or endorsed by the publisher.
